# The Impact of Graphene on the Fabrication of Thin Film Solar Cells: Current Status and Future Prospects

**DOI:** 10.3390/ma11010036

**Published:** 2017-12-27

**Authors:** Zhengqi Shi, Ahalapitiya H. Jayatissa

**Affiliations:** Nanotechnology and MEMS Laboratory, Department of Mechanical, Industrial, and Manufacturing Engineering (MIME), University of Toledo, Toledo, OH 43606, USA; Zhengqi.Shi@rockets.utoledo.edu

**Keywords:** graphene, thin film solar cell, efficiency, top contacts, back contacts

## Abstract

Commercial solar cells have a power conversion efficiency (PCE) in the range of 10–22% with different light absorbers. Graphene, with demonstrated unique structural, physical, and electrical properties, is expected to bring the positive effects on the development of thin film solar cells. Investigations have been carried out to understand whether graphene can be used as a front and back contacts and active interfacial layer in solar cell fabrication. In this review, the current progress of this research is analyzed, starting from the graphene and graphene-based Schottky diode. Also, the discussion was focused on the progress of graphene-incorporated thin film solar cells that were fabricated with different light absorbers, in particular, the synthesis, fabrication, and characterization of devices. The effect of doping and layer thickness of graphene on PCE was also included. Currently, the PCE of graphene-incorporated bulk-heterojunction devices have enhanced in the range of 0.5–3%. However, device durability and cost-effectiveness are also the challenging factors for commercial production of graphene-incorporated solar cells. In addition to the application of graphene, graphene oxides have been also used in perovskite solar cells. The current needs and likely future investigations for graphene-incorporated solar cells are also discussed.

## 1. Introduction

Graphene has attracted a great deal of attention in connection with nanotechnology since 2004 [1]. A perfect graphene sheet is only one-atom thick. Its atoms are arranged in a hexagonal structure, where atoms are all covalent bonded, which brings graphene superior mechanical strength (Young’s modulus exceed 1 TPa) [2]. After a decade’s intensive study, graphene has been already demonstrated with high carrier mobility (2 × 10^5^ cm^2^/V·s for suspended graphene) at the room temperature [3], great transparency (~2.3% light absorption across most ultraviolet (UV) and visible spectrum) [4], high thermal conductivity (~10^3^ W/m·K) [5] and high melting point (around 5000 K) [6]. Besides, the atom-layer structure also leads to a large surface area and great flexibility of graphene sheets. Thus, the low-cost graphene has been found in a wide range of applications, such as chemical sensors [7,8], medical devices [9], photo-detectors [10], energy storage [11], and manufacturing roll-to-roll electronic devices [12].

At present, a number of techniques have been developed for the fabrication of graphene. Besides the initial mechanical exfoliation of highly-organized graphite sheets [1], chemical vapor deposition (CVD) [13,14,15], supersonic spray [16], laser [17], and many other approaches have been established. Since graphene/graphene oxide (GO) is one-atom thick, conventional chemical synthesis that cannot be used for the production of graphene. Also, a substrate is necessary for growing graphene layers. Currently, the mainstream of graphene synthesis is still through the CVD method, which is popular in both academia and industries. In addition, the major synthesis path of GO and reduced graphene oxide (r-GO) is still the Hummer’s method [18] with different modifications on reaction conditions.

Solar energy conversion is another major area where graphene appears frequently. While the current photovoltaic (PV) market is still dominated by Si-based solar cells, thin film PVs that are based on CdTe [19,20], copper-indium-gallium sulfide (CIGS) [21], copper-zinc-tin sulfide (CZTS) [22,23], perovskite [24,25], and FeS_2_ [26] have been demonstrated with great potential. Lab-scale thin film PV devices have already reached 22% [24]. Thin film PV modules have also started the commercialization process and Si-based PV efficiency is getting closer to its theoretical upper limit [27]. On considering the high transparency, high carrier mobility, and conductivity, graphene has been tried as both contacts and interface layers in almost all kinds of PVs (e.g., Si, thin film, organic). In this review, we focused on the application of graphene on most commonly used thin-film PVs, with discussions on research advancement and the impact of graphene on those thin film PVs. The intention of this review is to improve scientific understanding of graphene properties that are relevant to PV applications and to accelerate the technological developments in the field.

## 2. Graphene and Graphene-Based Schottky Diodes

**Figure 1:**
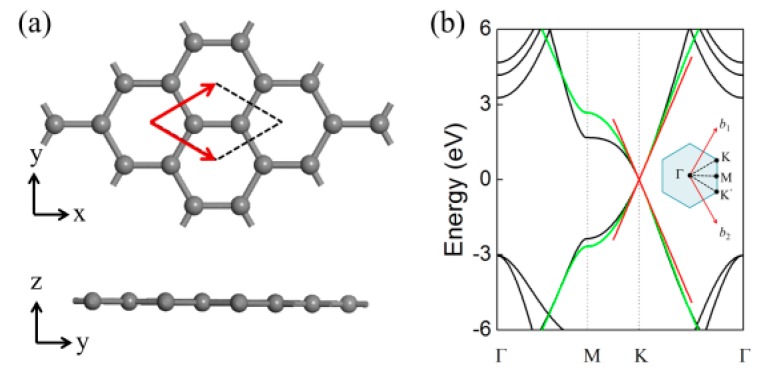
(**a**) Atomic structure and (**b**) energy bands of graphene. The energy bands have been calculated by first-principles (black line) and the tight-binding models: green lines indicate calculation assuming (*E*_0_ = 0) and red lines indicate cone-like band structure with linear dispersion near Dirac points K and K’ [35].

The microstructure of graphene consists of a repeatable two-dimensional (2D) hexagonal lattice. Each peak position is occupied by carbon atom, with another equivalent three covalent-bonded nearest neighbors, as shown in Figure 1a, where the corresponding primitive lattice vectors aree also labeled. Thus, the reciprocal lattice and the hexagonal Brillouin zone could be inferred, as shown in Figure 1b. together with the band structure of graphene. It is clear that the conduction and valence bands are attached at the two crystal momentum corners of the hexagonal Brillouin zone. Thus, the band gap of primitive graphene is zero (at K-K’), which is shown in Figure 1b, and graphene could be considered as a semi-metal. 

When in contact graphene with a semiconductor, a Schottky junction would be formed. However, the Fermi level of graphene could be easily shifted by chemical doping or carrier injection under a bias [28,29]. Therefore, unlike the typical metal/semiconductor Schottky junction, the graphene/semiconductor could have a modified Schottky barrier height. Some early-stage experimental results claimed the formation of Schottky diode on various substrates, such as Si [30,31], GaAs [32], and SiC [33]. A strong rectifying behavior could be observed from their current-voltage (I-V) characteristics. Further calculation that is based on capacitance-voltage (C-V) measurement gave an estimation of graphene work function to be in the range of 4.4–4.8 eV [34].

**Figure 2:**
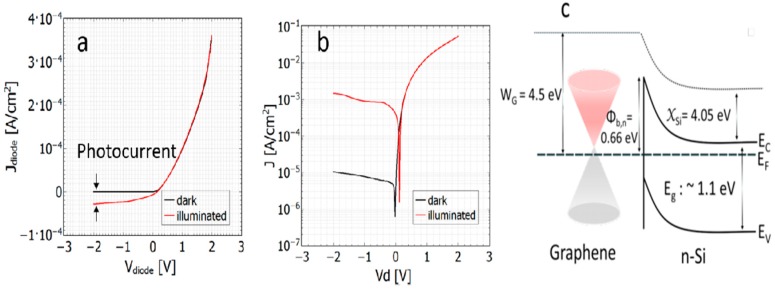
J-V plot of the graphene/n-Si diode on (**a**) linear and (**b**) semi-logarithmic scale in the dark and under illumination; (**c**) the graphene/n-Si interface at zero bias voltage. *E_C_*, *E_V_*, *E_F_*, *E_g_*, *W_G_*, *χ_Si_* and *Φ_b_* indicate conduction band, valence band, Fermi level, bandgap, graphene work function, Si electron affinity and Schottky barrier height, respectively [36].

Moreover, Riazimehr et al. [36] measured a photocurrent of graphene/n-Si Schottky diode and observed a light-generated current as shown in Figure 2a, indicating the photogeneration of electron-hole (e-h) in Si and the carrier extraction ability of graphene. Further investigation of mapping the position of incident light at a different location resulted in an effective load across the junction due to the different photocurrent with the corresponding light incident position. The resulting current–voltage characteristics exhibit rectifying diode behavior, with a barrier energy of 0.41 eV on n-type Si and 0.45 eV on p-type Si at the room temperature. Since Si is the most typical sunlight absorber, the contact nature of graphene with other thin-film PV materials could follow the similar behavior, as seen in Figure 2b. Also, energy band diagram of Graphene/n-Si is shown in Figure 2c.

## 3. Research Progress of Graphene-Composited Thin Film Solar Cell

### 3.1. Graphene with CdTe

**Figure 3:**
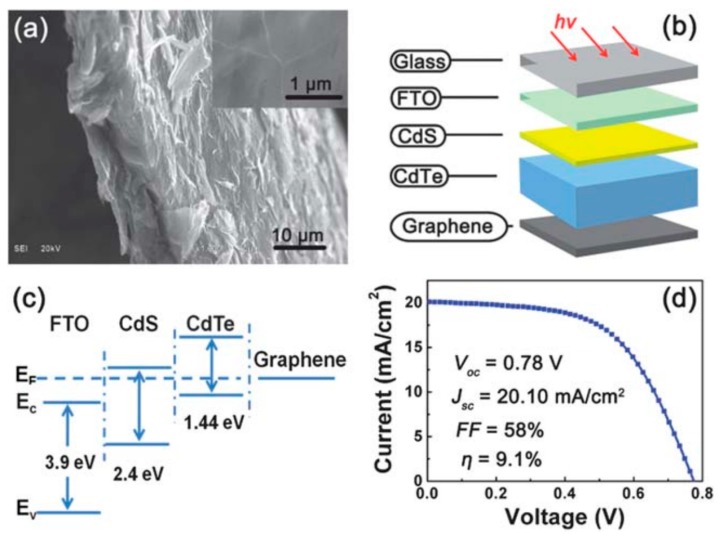
(**a**) Scanning electron microscopy (SEM) of a graphene incorporated CdTe film, insert figure was the top-view. (**b**) A schematic draw of 3D graphene-incorporated CdTe solar cell. (**c**) Band structure of the CdTe solar cell with graphene back contact. (**d**) J–V curve of CdTe solar cell with graphene back contact [39].

With high transparency, carrier mobility and flexibility, graphene was first considered to be the alternative contact material. To the best of our knowledge, the first successful graphene-encountered CdTe device was reported by Bi et al. [37] with consideration of similar work function between graphene and fluorine doped tin oxide (FTO) [38]. In their work, the graphene was synthesized on a Cu foil by an atmospheric pressure CVD (APCVD) and later transferred to a glass substrate. By altering the H_2_ flow rate, the number of graphene layers could be well controlled, which also provides a wide selection range of transparency and sheet resistance. Two devices, with and without ZnO blocking layer, were fabricated and characterized. The device with ZnO layer showed a higher efficiency (4.17%) than the one without ZnO (2.81%), indicating the importance of ZnO: a good contact with graphene and current leakage protection. Followed by their initial work, this group reported a great advantage of the graphene-CdTe device with graphene as the back contact. In order to provide a better coverage and contact on CdTe, the graphene was synthesized with a three-dimensional (3D) structure using porous Ni foam as the growth substrate. A similar method was employed to grow graphene and the 3D structure was successfully observed and transferred to CdTe device. The final graphene back contact thickness exceeded 10 µm with an excellent electrical conductivity (550–600 S/cm), which assisted a significant device efficiency improvement up to 9.1%, as shown in Figure 3. Moreover, with a relatively large active area (1.0 cm^2^), this work indicated the possibility of large-scale device fabrication [39].

Moreover, graphene-based composite back contact was also tried: Liang et al. [40] reported the usage of Cu nanowire/graphene back contact for CdTe solar cell. Their graphene was also prepared with similar method [39], and then mixed with hydrothermally synthesized Cu nanowires. The mixture was brushed onto the CdTe layer and the device exhibited an efficiency of 12.1%, which, according to their comparison, was higher than single Cu and graphene back contact (see Table 1). This result might be related to a better hole collection process of graphene 3D network and the formation of the ultrathin CuTe interface layer. However, with an extra heating process on the device, the efficiency was decreased due to the lower thermal stability of graphene contact. It is important to prepare graphene layer without the interfering of oxidation species. There is no indication of any reactivity between Te and carbon, while Cu has the lowest solubility in carbon, which prevents the formation of any oxide layer. Thus, a decrease of efficiency of annealed device could be attributed to the interference of oxygen in graphene/CdTe interface.

**Figure 4:**
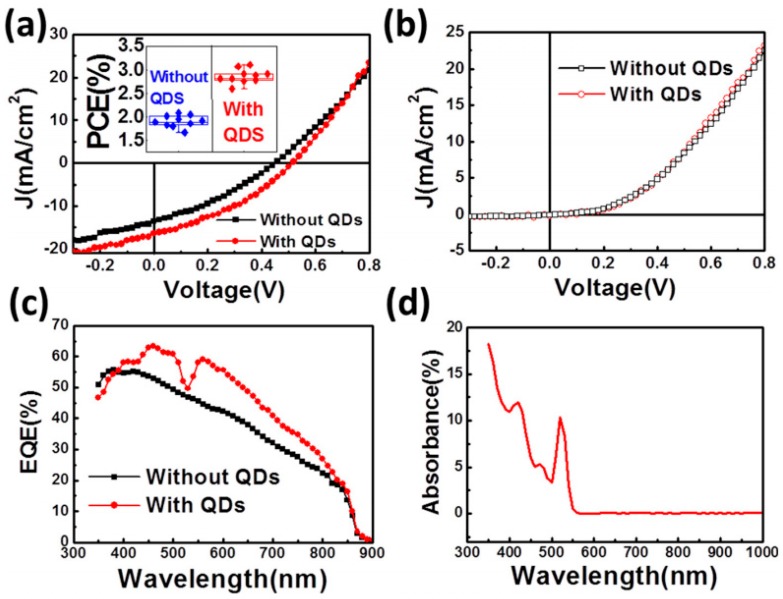
Current density versus voltage (J-V) characteristics of the graphene added devices with and without CdSe QDs under (**a**) AM1.5G illumination and (**b**) dark. The inset of (**a**) shows statistical results of devices with and without QDs. (**c**) External quantum efficiency (EQE) curves of the devices with and without CdSe QDs. (**d**) The absorption spectrum of the CdSe QDs [43].

In addition, impurity doping into graphene was also tried in back contacts. One example is the comparison among boron-doped graphene, intrinsic graphene and reduced graphene oxide (r-GO) [41]. These results showed the boron-doped graphene back contact was the best choice due to the highest conductivity. This could be due to extra holes introduced into graphene through boron doping during the initial graphene synthesis. In addition to the efforts on applying graphene as the front and back contacts, trials of using graphene to replace CdS inside the CdS/CdTe system were also processed. Ideally, the work function of graphene is close to the electron affinity of CdTe, indicating a possible junction formation. According to Brus et al. [42], their graphite/n-CdTe (fabricated with the Cd-rich atmosphere) was able to acquire an efficiency of 1.36%, with a clear shunting effect. Later, Lin et al. [43] announced an unusual substrate-configured graphene/CdTe device. In this work, the graphene was synthesized by a CVD process and was transferred on to both CdTe and patterned SiN_x_, which served as a separator between Ag top grid and CdTe. Their device acquired a relatively high efficiency of 2.08%. Moreover, to test the importance of original window layer CdS/CdSe, a separately-prepared CdSe quantum dots was prepared and coated onto the graphene supporting layer, which elevated the efficiency result to 3.1%. Those extra n-type quantum dots also contributed to light-generated electron collecting, which led to a clear increase in both short-circuit current density (J_sc_) and external quantum efficiency (EQE), as shown in Figure 4.

In addition to the above experimental results, simulation work also indicated the great potential in the application of graphene in CdTe system. Aldosari et al. [44] established a model with graphene at both front and back contact. They claimed that graphene front contact, with lower thickness, would lead to less incident current loss. Thus, mono-layer graphene would be the best choice. Also, the combination with ZnO would significantly improve the overall conductivity. For the back contact case, besides the proper work function of graphene, its appearance also made the double-sided illumination possible for CdTe solar cell. Therefore, the J_sc_ would be greatly enhanced and the CdTe thickness could also be reduced to around 1 µm to minimize unnecessary carrier generation. This group also claimed that the dangling bonds between graphene networks might generate movable ions, especially with the co-existence of metal dopant. Those free ions would diffuse into the p-n junction interface, and, therefore, cause damage to the device rather than at the back contact interface [45]. In addition, several reports related to the application of single-wall carbon nanotube were also indicated with a better device efficiency up to 14.1% [46]. More detailed information could be available from another review [47].

Although graphene was already demonstrated with great transparency and conductivity, the actual practice showed that more work is required to optimize the growth conditions, such as growth atmosphere profile and metallic doping, as well as their effects on graphene contact properties. Also, understanding of the thermal stability of graphene and graphene composite is also necessary. Overall, that reported progress showed that graphene could be a suitable replacement for FTO and metal back contact for CdTe system with a lower cost and easy manufacturing.

### 3.2. Graphene with CIGS

**Figure 5:**
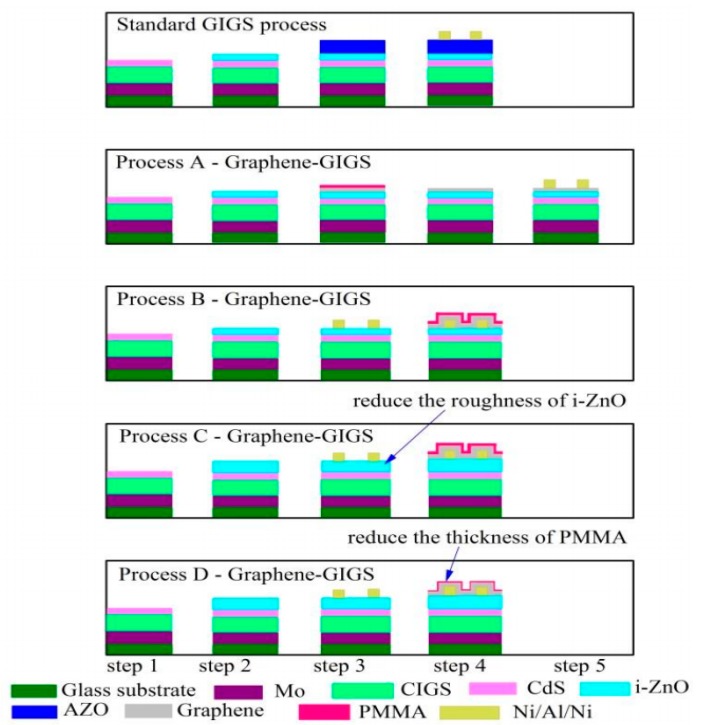
Schematic of multilayer arrangement in possible graphene-based CIGS device [48]. Graphene is incorporated at step-4 in all processes except standard process.

Similar to the CdTe system, graphene was first considered as an alternative front contact. Yin et al. [48] first reported an effort of replacing AZO with graphene or graphene/PMMA composite. Their results showed that the direct contact between graphene and i-ZnO was poor due to surface damage during PMMA removal, which also affects carrier collection through evaporated metal grids. Thus, they optimized the fabrication process, as shown in Figure 5 [48]. In this study, all possible CIGS solar cells have been described and the graphene has been incorporated at step-4 in all of the processes, except for standard process. Finally, the sheet resistance was minimized and they acquired a significant efficiency increase from the initial 0.43% to 13.5% as listed in Table 2. Those devices also showed better optical properties than the reference. However, the best result still had a small gap with their reference copper indium gallium sulfide (CIGS) device fabricated with AZO layer (Eff. 14.9%) probably due to a lower sheet resistance. They also believe that the graphene/PMMA could be a moisture protection of the CIGS solar cell.

Moreover, the Brookhaven National Lab achieved a direct n-doped graphene/CIGS Schottky junction [49]. The strong n-type doping on graphene was accomplished through Na diffusion from soda-lime glass (SLG) substrate through CIGS grain boundaries, which is similar to the ion migration in CdTe layer. When under standard AM 1.5G light, this device displayed a high photocurrent and a typical rectifying behavior. The J_sc_ was about 13 mA/cm^2^ and the efficiency was around 1%. However, the strong Na diffusion would affect the p-type region, which may affect the built-in potential and the open-circuit voltage (V_oc_) of the device.

According to the literature, very limited investigations have been carried out regarding the successful application of graphene as the back contact for CIGS although the work function requirement was similar to CdTe. But, a simulation work based on refractive index and extinction coefficient indicated that the graphene back contact would lead to a higher J_sc_ and efficiency than the front contact case [50]. As predicted in the CdTe model, the high-transparent graphene contact would enable double-side illumination on the solar cell, which may create efficiency that similar to the dominant Si-based solar cells. The successful 3D dimension of graphene back contact on CdTe might be a good reference for developing similar layer for CIGS.

At present, no experimental record shows the effect of graphene on copper-zinc-tin sulfide (CZTS) system. Only a simulation result predicted the advantage of using graphene front contact, rather than the conventional indium-tin-oxide (ITO) [51]. In their work, such replacement was believed to increase all of the solar cell parameters, as well as further minimizing the CZTS absorber thickness. In addition, researchers also demonstrated the successful synthesis of copper indium sulfide (CIS) films on graphene contacts. The CIS films fabricated on graphene layers, rather than on Mo contacts, showed a strong preferential growth in (112) direction. A strong ohmic contact was also identified [52]. Another study focused on copper tin sulfide (CTS) synthesis on r-GO and their results showed that CTS/r-GO could be a great photo-conductor. With the addition of ethylenediaminetetraacetic acid as the complexing agent during initial synthesis, the photocurrent could be enhanced by 60% [53]. Those discoveries indicate a great potential of graphene application on the photo-sensitive ternary phases of CIGS and CZTS.

The above information, it could be inferred that the application of graphene on CIGS or its relevant materials system is still in its early stage. Similar to CdTe case, more understanding and experience are required for the optimization of transport phenomenon at the GIGS/graphene interface. Based on the current literature, it can be expected that the performance of CIGS solar cells will be further enhanced by the application of graphene.

### 3.3. Graphene with Perovskite

In the past few years, the organic-inorganic lead-halide perovskite solar cells (PSCs) achieved fast efficiency improvement from 3.8% to 22.1% [54]. The most typical perovskite light absorber, CH_3_NH_3_PbX_3_ (X = Cl, Br, I), had been demonstrated with a suitable band gap and high absorption coefficient [55]. The device was first found during research on dye sensitized solar cell (DSSC). However, since a later investigation identified the pathway of light-generated carriers, which were separated through different charge transport layers and then be transferred into the contact, a number of efforts had been made to identify the possible candidates of electron transport layer (ETL) and hole-transport layer (HTL). Among them, TiO_2_ and Spiro-OMeTAD (2,2′,7,7′-tetrakis (*N*,*N*-di-pmethoxyphenylamine)-9,9′-spirobifluorene) are the most widely-used ETL and HTL, respectively [56]. DSSC devices that are based on planar thin film design had been successfully implemented [57,58]. Those charge transport layers also served as a protection of the core part between them, since perovskite materials could be degraded soon with exposure to moisture and/or air. Thus, the purpose of graphene becomes not only as the front/back contacts, but also the ETL and HTL layers could be also partially or totally replaced with graphene, r-Go or other graphene-related carbon materials. The following section describes most relevant and appropriate research on these topics.

Graphene can be used to improve either or both of charge injection and collection at the electrodes. Thus, the power conversion efficiency and durability can be enhanced. This can alleviate the problematic carrier recombination processes, which are known to increase due to the material instability at the interfaces of soft materials in PSCs. When free charges are fast injected from perovskite to the electron transport layer, the degradation of perovskite and non-radiative recombination are reduced. Subsequently, mixing of mesoporous TiO_2_ with graphene and lithiated graphene have been successfully employed to enhance both PCE and stability of PSCs [59]. More investigations are required in this field to further enhance the performance of PSCs using graphene.

#### 3.3.1. Graphene in ETL

**Figure 6:**
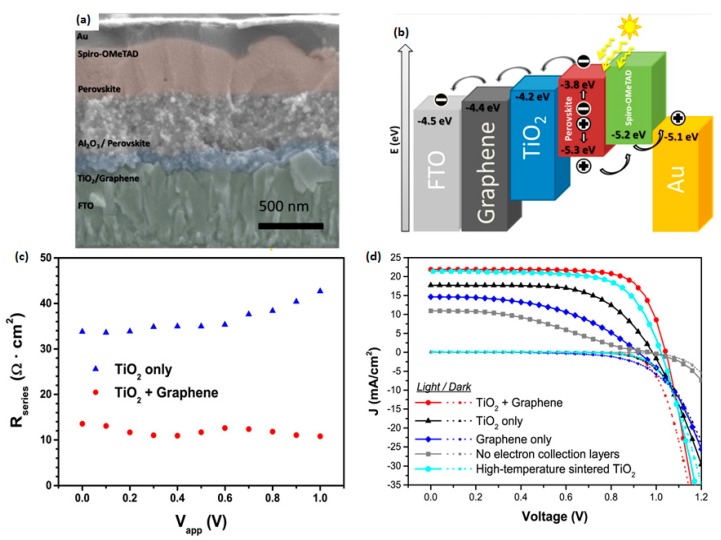
(**a**) Cross-sectional SEM image and (**b**) energy levels of the PSC with a graphene-TiO_2_ composite as the electron transport layer (ETL). (**c**) The series resistance of the perovskite solar cells (PSCs) with a graphene-TiO_2_ composite or TiO_2_ only as the ETL. (**d**) J-V characteristics of the PSCs with different electron collection layers under AM 1.5G and dark [60].

The first reported graphene-enhanced PSC was accomplished through graphene/TiO_2_ composite as the ETL [60]. Their graphene was prepared through multiple sonication, centrifugation, and re-dispersion, after the initial sieving. The percentage of graphene in the mixture paste was controlled by the volume of graphene. After the device fabrication, they found that a small portion of graphene could enhance the electron collection due to a higher carrier mobility and work function matching between TiO_2_ and FTO. Their highest efficiency was 15.6% when compared with typical high-temperature-annealed TiO_2_ (14.1%). A detailed J-V curve comparison was shown in Figure 6. However, more graphene incorporation cannot further improve the efficiency due to possible recombination at the interface of graphene/perovskite. Their data showed that the suitable range for graphene is 0.6–0.8 wt %. Moreover, graphene also reduced the annealing temperature of the TiO_2_ layer. Those graphene-involved devices had a TiO_2_ annealing temperature of 150 °C, rather than the typical 500 °C, making the fabrication process less costly. Zhu et al. [61] reported the usage of graphene quantum dots in between TiO_2_ and perovskite. This insertion minimized the electron extraction time from the perovskite, and, therefore, the efficiency was enhanced from 8.81% to 10.15%.

#### 3.3.2. Graphene Oxide in ETL

**Figure 7:**
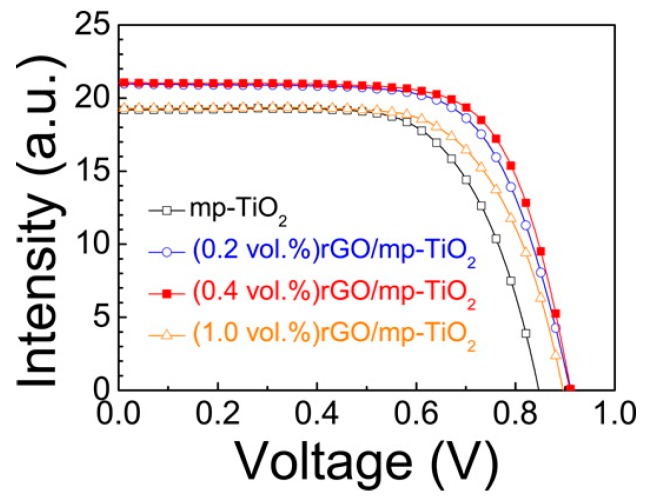
J-V curve of rGO/ mesoporous-TiO_2_ nanocomposite based perovskite solar cell with varying rGO contents (0.2 vol %, 0.4 vol %, and 1.0 vol %). The black line stands for the reference cell with mesoporous-TiO_2_ nanolayers [62].

Also, GO and r-GO were investigated to boost the efficiency of PSCs. The r-GO synthesized by Hummers’ method was mixed with TiO_2_ slurry to form the final mixture and spin coated in a TiO_2_ blocking layer [62]. By altering the volume percentage of r-GO in the total ETL slurry, the r-GO concentration could be well modified, and, similarly, only a small portion of r-GO has a positive effect on the device performance. This is due to the r-GO light absorption in the visible light area. With an increased amount of r-GO, the incident light that was acquired by perovskite was reduced. Therefore, the J_sc_ was cut down. According to their results, the 0.4 vol % was the best ratio, with an efficiency of 14.5%, which was clearly higher than their TiO_2_-only device (11.5%). The corresponding J-V characteristics are shown in Figure 7. Another report using r-GO in both compact and mesoporous TiO_2_ also showed an increased efficiency with its reference cell. However, this work showed a lower efficiency of 9.3%, with an r-GO ratio of 0.15 wt % in compact TiO_2_ and 0.015 wt % in meso-TiO_2_ [63]. Besides the mixed slurry approach, the layer-by-layer method could be also effective and its experimental proof was reported in 2016 [64]. A modified Hummers’ method [59] was used for GO synthesis. Li doping was applied inside the re-dispersed GO solution, and the total mixture was re-dispersed by water/ethanol that was ready for spin coating under an inert atmosphere. By inserting this Li-doped GO layer, the J_sc_ and fill factor (FF) was boosted but the V_oc_ had a slight decrease due to TiO_2_ conduction band edge shrinking (negative effect of Li doping). The efficiency of the GO-injected device was 11.8% and the reference cell was 10.3%. Moreover, the GO layer injection significantly reduced the J-V hysteresis. According to the investigators, it is believed that Li-GO layer could reduce the trap-assisted recombination at TiO_2_/perovskite interface. The addition of Li-GO layer also reduced the device decay rate in the initial stage, but has no clear improvement for the long-term device stability.

#### 3.3.3. Graphene and Graphene Oxide in HTL

**Figure 8:**
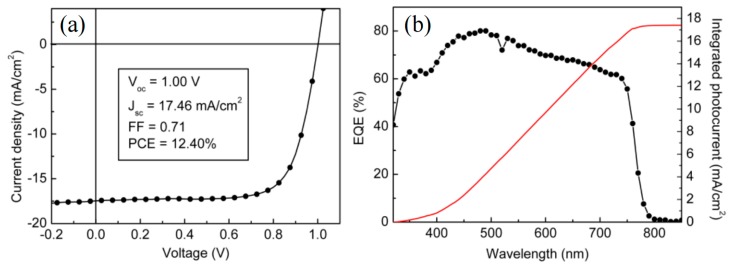
(**a**) J–V characteristics and (**b**) EQE spectrum of the champion device with graphene/graphene oxide (GO) layer [65].

Research on using graphene-related films as HTL also attracts great attention. Wu et al. [65] spin-coated GO films that were prepared from a modified Hummer’s method, as described in a previous protocol [66], on ITO substrate as the HTL. The perovskite film grown on GO showed better film coverage and larger crystal size than typical PEDOT:PSS and bare ITO. Also, a strong preferential growth in (110) plane was observed for perovskite on GO layer, indicating high carrier mobility in perovskite films. Thus, the carrier separation and J_sc_ could be enhanced. The inverted PSC with GO as the HTL achieved a high PCE of 12.40%, as shown in Figure 8, while the one with PEDOT:PSS showed only 9.26%. They also suggested that a higher GO suspension concentration and thicker GO film could have a better enhancement with perovskite, and, hence, increased device efficiency. 

Different attempts were made to accomplish this new HTL. The performance of r-GO was compared with the most typical Spiro-OMeTAD as the hole-transport layer [67]. The GO layer was spray-coated under N_2_ atmosphere on a pre-heated perovskite layer. In addition, the reference samples had either a spin-coated GO layer from the same GO suspension or a Spiro-OMeTAD layer. After evaporating the top Au grid, the J-V characteristics of PSCs showed that the Spiro-OMeTAD had a greater device efficiency due to both kinds of GO layers, and very little statistical difference could be found between the device efficiency of spin-coated and sprayed GO-based PSCs. However, due to a low stability of Spiro-OMeTAD, the efficiency of PSCs with Spiro-OMeTAD suffered a 41% loss after 1987 h. In the meantime, PSCs with GO showed a significantly good stability, and their efficiency increased from 4.87% to 6.62%, which was a 36% efficiency improvement due to better prevention on photo-generated carrier recombination at GO layer than Spiro-OMeTAD [68]. Detailed comparison on device stability could be found in Table 3.

**Figure 9:**
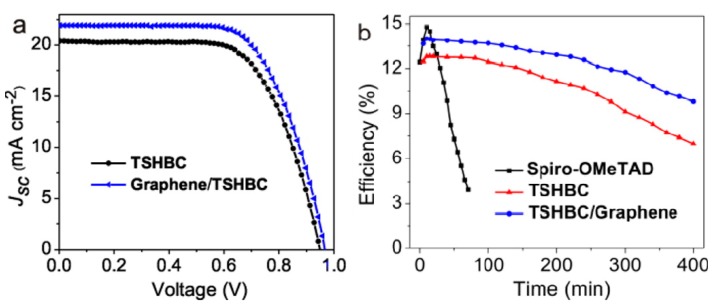
(**a**) Best I−V curves of TSHBC (perthiolated trisulfur-annulated hexa-peri-hexabenzocoronene) and G/TSHBC-based cells and (**b**) Device stability test with AM1.5G under 45% humidity [69].

A similar comparison was also observed by Cao et al. [69]. Moreover, the initial device efficiency was also greatly improved in this work: A functionalized nano-graphene hole-transport material (perthiolated trisulfur-annulated hexa-peri-hexabenzocoronene, TSHBC) was applied. The thiol group, according to them, could be a strong binder between perovskite and graphene. Therefore, the hole-transportation rate would be accelerated. They reported an efficiency of 12.81% with a device structure of FTO/TiO_2_/CH_3_NH_3_PbI_3_/TSHBC/Au. Moreover, due to a better chemical stability of graphene, the fabrication process was finished in the air with 45% humidity, which was a bad condition for Spiro-OMeTAD fabrication. This group further improved their device efficiency up to 14% with introducing extra graphene sheets into TSHBC layer to improve its hole transportation phenomenon. Also, with a higher water contact angle of TSHBC/graphene than Spiro-OMeTAD, the 14% PSCs could gain a better moisture-resistance, which could also extend the device lifetime as shown in Figure 9. Another work from Hong Kong provided a detailed measurement of the hole extraction rate in both single and multilayer graphene hole transport layer, which was 3.7 and 5.1 ns^−1^, respectively [70]. Their best cell reached an efficiency of 11.5% with the help of multilayer graphene. The graphene HTL, according to their results, formed a Schottky barrier at the perovskite/graphene interface. Thus, the hole extraction and transportation processes could be enhanced while electrons could be blocked and recombination rate was decreased as well.

The investigations were conducted not only on the single graphene/GO HTL but also on the graphene/GO-composited HTL. In 2013, Luo et al. [71] claimed a 13.01% PSC using iodide-reduced GO together with Spiro-OMeTAD as the HTL. After initial GO preparation from both Hummer’s and Offeman’s method, the GO suspension was mixed with the self-prepared FeI_2_ solution and reduced by HCl. The r-GO and Spiro-OMeTAD were sequentially spin-coated on perovskite. Since r-GO and Spiro-OMeTAD have different energy levels, holes generated in perovskite could flow into either of them. Reduced GO could also extract holes from lower-level Spiro-OMeTAD and bring them to Au contact. Thus, this combined HTL works more efficient than either of them. Also, the existence of r-GO greatly improved the wettability and stability of PSCs. Another composited HTL, GO/PEDOT:PSS, brought an efficiency of 9.7% while the PSCs with PEDOT:PSS and GO showed only 8.2% and 6.4%, respectively [72]. The composited HTL led to the highest shunt resistance (R_sh_) and lowest sheet resistance (R_s_). The addition of graphene improved the device stability compared with bare PEDOT:PSS. In turns, PEDOT:PSS improved the surface coverage of ultrathin graphene.

#### 3.3.4. Graphene as Contacts

**Figure 10:**
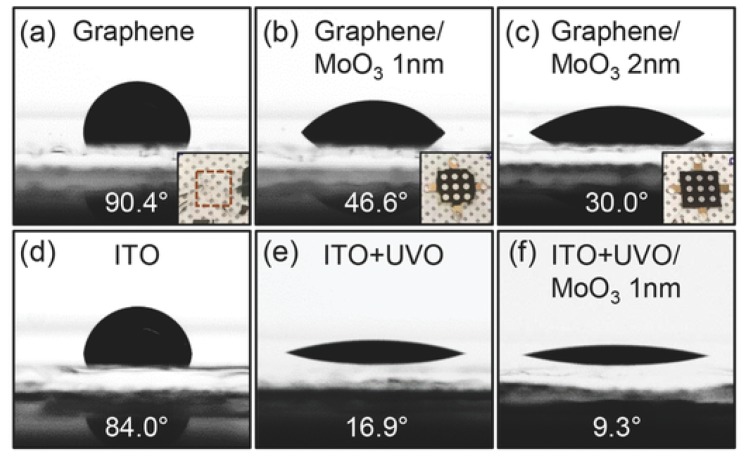
PEDOT:PSS droplet contact angles on (**a**) as-prepared graphene, (**b**) graphene covered with 1 nm MoO_3_, (**c**) graphene covered with 2 nm MoO_3_, (**d**) as-prepared ITO, (**e**) UVO-treated ITO, (**f**) ITO covered with 1 nm MoO_3_ after UVO treatment. The insets in (**a**–**c**) showed the optical images of PEDOT:PSS/MAPbI_3_ films fabricated on the corresponding glass/graphene surfaces [74].

**Figure 11:**
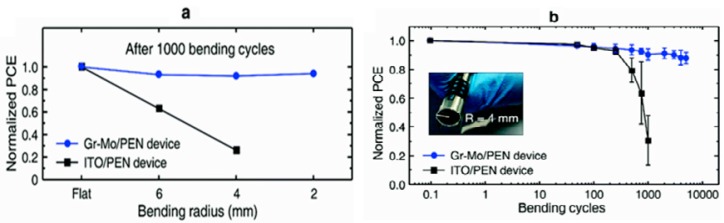
(**a**) Normalized PCEs of the Gr-Mo/PEN and ITO/PEN devices measured after 1000 bending cycles with bending radius of flat, 6, 4, and 2 mm. (**b**) Normalized PCEs vs bending cycles at a bending radius of 4 mm for the Gr-Mo/PEN and ITO/PEN devices. The inset photograph shows the actual bending situation [75].

Although application of graphene in ETL and HTL was the primary focus, graphene could also be served as the electrodes of PSCs due to controllable conductivity, great carrier mobility and transparency. PSCs equipped with graphene electrode could be able to accept light from both sides. As reported by You et al. [73], the CVD-synthesized graphene was transferred with PMMA on a thin layer of PEDOT:PSS, which helped to modify the conductivity of thin-layer graphene electrodes. Graphene accumulation would also decrease the sheet resistance but the transparency would be also decreased. Their best PSC was with 2-layer graphene electrodes and showed an efficiency of 12.02% and 11.65% with illumination from FTO and graphene sides, respectively. Sung et al. [74] fabricated graphene on an inverted PSC with similar conductivity assistance. But they inserted a thin MoO_3_ layer between graphene and PEDOT:PSS due to better wettability of PEDOT:PSS on graphene/MoO_3_ shown in Figure 10. The device characterization showed 2 nm MoO_3_ layer had an improvement for both graphene and ITO electrodes: The best graphene-based PSCs showed an efficiency change from 12.1% to 17.1%, while the best ITO-based devices showed 17.6% and 18.8%, respectively. Table 4 showed device parameters for all PSCs. Detail characterization showed the MoO_3_-composited graphene had a higher work function of 4.71 eV. Also, the graphene sheet resistance dropped down by 5 folds with 2 nm MoO_3_. These results explained the clear efficiency change. Recently, Yoon et al. [75] fabricated a flexible PSC by using similar electrode design. The PSC had an inverted configuration with the best efficiency of 16.8%, while the flexible ITO electrode acquired 17.3%. However, after 1000 bending cycles, the ITO-based PSCs showed drastic efficiency decrease while the graphene-based PSCs suffered only minor change. Compared with ITO or other metal electrode, graphene electrodes occurred no crack after long-term bending, which showed great potential in flexible PSCs’ fabrication. The detailed comparison could be found in Figure 11. 

#### 3.3.5. Other Applications of Graphene in PSCs

In addition, graphene could also be mixed with perovskite slurry before depositing the adsorption layer. Hadadian et al. [76] applied an N-doped r-GO into perovskite slurry and they found an increase of perovskite grain size due to a slower crystallization. The graphene incorporation also brought surface passivation at perovskite/Spiro-OMeTAD interface due to the excellent carrier separation capability of graphene. Their best device had an efficiency of 18.7%, which is by far the best performance among all the graphene-based PSCs. Also, by combining the advantage of graphene at both ETL and HTL, one group acquired an efficiency of 18.2%, which was already close to those top-tier PSCs [77]. However, the 16 h illumination test revealed nearly 70% efficiency loss and a further 1000 h shelf-life condition test displayed about 45% efficiency loss, indicating the graphene-composited interface may not be as stable as predicted.

Overall, graphene has been demonstrated with sufficient space in PSCs. With the proper enhancement of conductivity and surface compatibility, graphene could bring PSCs to the next stage. The soft nature of graphene also provides possibility to produce flexible PSCs, which could be identical to large-scale and roll-to-roll production.

## 4. Applications and Future Research

The above research indicates that the graphene can be used for enhancing the performance of solar cells in various ways. Firstly, graphene can be used in top and back contacts of CdTe solar cells due to its work function matching with CdTe and a better carrier mobility. However, the top contact needs 1–5 layers of graphene in order to minimize the optical absorptions. Due to the zero bandgap of graphene, a schottky junction would be formed between graphene and CdTe, however; no evidence supports that this kind of junction improves carrier separation process. Similarly, graphene is also applied in graphene-composited CIGS or CZTS system (to replace AZO and/or combine with Mo or other metal contacts) but additional efforts could be made on reducing the contact resistance between graphene and other components (i-ZnO, top grid and/or absorber) in CIGS and CZTS based cells. In PSCs, graphene could be applied in every component: in ETL, graphene was initially mixed with TiO_2_ but Li-GO/TiO_2_ double-layer structure was also proved successful. Bu considering the advantage of graphene over FTO, a new ETL/contact structure with graphene and graphene-incorporated ETL might be considered, however; graphene concentration should be maintained at a low level. In HTL fabrication, graphene/GO could be used as HTL by itself or mixed with some typical hole-transport materials. However, the key is to find an efficient structural and electrical binder between graphene/GO and perovskite otherwise the PEC may not be improved. Such binder is also important with graphene front/back contacts in PSCs. In addition, more work could be focused on introducing graphene into perovskite layer due to possibly better perovskite crystal formation, which is vital to improve device efficiency and durability. Table 5 summarizes the key fields towards to those findings and suggestions. Authors expect more investigations will be performed in CZTS-based solar cells in the future. 

Since the solar cells are large-area devices, process scalability is very important for a commercial viability of usage of graphene in solar cells. In particular, the top contacts need 1–5 layers of graphene and it is a challenging process to manufacture such materials in large-scale. Also, one of the primary reasons for degradation of PSCs is the heating effect, which produces photo-inactive phase in perovskite layer. Since graphene is one of the highest thermal conductive materials, the heating effect can be significantly controlled by an appropriate level of incorporation of graphene. These research can be very useful steps towards increasing the durability of PSCs. Many investigations indicated that the pristine graphene can be used in solar cells. However, further improvements of conversion efficiency might be possible with functionalized graphene. Therefore, investigations related to the application of functionalized graphene in the solar cells can be very useful in both fundamental and application viewpoints. Also, further investigations are important to understand the application of graphene as front and back contacts of flexible and semitransparent solar cell system.

## 5. Conclusions

Graphene, with its extremely high carrier mobility and transparency together with suitable work function, had been proved as a suitable replacement of the TCO layer for CdTe and CIGS system. The graphene front contacts could have a mono-layer structure but the back contacts should have adequate thickness to prevent extra current loss. However, the experiment indicated that the graphene layer itself tends to suffer incomplete surface coverage and a higher sheet resistance in a lateral direction, which leads to a current leakage and insufficient carrier transportation. Suitable metallic doping could improve the performance of device. In order to have advantage of band structure, the controllability of number of layers in a graphene manufacturing should be optimized.

For the perovskite solar cells (PSCs), a positive effect has also been demonstrated by application of graphene in almost every component. Compared with the conventional design, the usage of graphene enhanced the carrier extraction and device stability (compared with Spiro-OMeTAD). The flexible devices is also achieved by the fabrication of printable graphene-based PSCs on a soft substrate. Also, the preliminary test showed such flexible cells were durable after a large amount of bending cycles. Moreover, the application of graphene in multiple positions already showed a higher efficiency than PSC without graphene. However, the feasibility studies are necessary to integrate graphene usage in the solar cell manufacturing processes. The application of r-GO in PSCs was also briefly discussed. Although the long-term stability test showed the advantage of graphene over the conventional HTL, the loss of device parameters should be addressed before the commercialization stage. 

## Figures and Tables

**Table 1:** Device performances of the CdTe cells with four different back contacts [40]. This table contains conductivity (σ), carrier mobility (μ), open circuit voltage (V_oc_), short circuit current density (J_sc_), fill factor (FF) and efficiency (Eff) for back contacts made with Cu thin films (Cu TFs) followed by a thick Ni layer, graphite or graphene paste containing Cu particles (Cu Ps, ≈75 μm in diameter) and copper nanowires (Cu NWs)/graphene.

Back Contact Material	σ [S cm^-1^]	μ [cm^2^ V^-1^ s^-1^]	V_oc_ [mV]	J_sc_ [mA cm^-2^]	FF [%]	Eff. [%]
Cu NWs/Graphene	16.7	16.2	801	22.4	67.4	12.1
Cu Ps/Graphene	14.2	13.2	805	21.3	68.1	11.7
Cu Ps/Graphite	5.5	5.0	790	21.2	62.5	10.5
Cu TF	-	-	740	21.1	58.0	9.1

**Table 2:** Graphene-composited copper indium gallium sulfide (CIGS) cell performance [48].

Samples	TCE	Process [Thickness of i-ZnO]	V_oc_ [mV]	J_sc_ [mA/cm^2^]	Fill Factor [%]	PCE [%]
#1	150nm AZO	Reference [50 nm]	601	32.8	74.3	14.9
#2	1-layer G	Process A [50 nm]	543	0.93	25.3	0.43
#3	400nm PMMA/1-layer G	Process B [50 nm]	595	23.5	40.4	5.63
#4	400nm PMMA/4-layer G	Process B [50 nm]	596	27.4	44.2	7.20
#5	400nm PMMA/4-layer G	Process C [125 nm]	595	31.2	56.3	10.5
#6	400nm PMMA/4-layer G	Process C [200 nm]	603	30.1	52.2	9.50
#7	150nm PMMA/4-layer G	Process D [125 nm]	601	32.4	69.1	13.5

**Table 3:** Device stability test results (1987 h) for both kinds of PSCs (perovskite solar cells) [68].

HTM	V_oc_ [V]	J_sc_ [mA cm^−2^]	FF [%]	PCE [%]	PCE Relative Variation [%]
Spiro-OMeTAD As-Prepared	1.02	15.70	68.78	11.06	−41
Spiro-OMeTAD 1987 h	1.00	10.50	61.75	6.50	
RGO As-Prepared	0.91	8.95	59.78	4.87	+36
RGO 1987 h	0.95	11.5	60.54	6.62	

**Table 4:** Device parameters of PSCs with graphene/MoO_3_ and indium-tin-oxides (ITO) electrodes [74].

Sample ID	Electrode	MoO_3_ Thickness [nm]	V_oc_ [V]	J_sc_ [mA cm^-2^]	FF	PCE [%]	Best PCE [%]
G-M1	Graphene	1	0.72 ± 0.36	17.6 ± 6.3	0.45 ± 0.09	6.7 ± 4.2	12.1
G-M2		2	1.03 ± 0.02	21.9 ± 0.4	0.72 ± 0.02	16.1 ± 0.6	17.1
G-M4		4	1.00 ± 0.01	22.9 ± 0.4	0.70 ± 0.02	15.9 ± 0.5	16.2
ITO-M0	ITO	0	0.96 ± 0.01	21.4 ± 0.5	0.83 ± 0.02	17.0 ± 0.4	17.6
ITO-M1		1	0.97 ± 0.01	22.6 ± 0.4	0.83 ± 0.01	18.2 ± 0.5	18.8
ITO-M2		2	0.95 ± 0.01	22.2 ± 0.4	0.76 ± 0.01	16.1 ± 0.4	16.9
ITO-M4		4	0.94 ± 0.01	21.0 ± 0.4	0.74 ± 0.01	14.7 ± 0.6	15.7

**Table 5:** Findings and suggestions of graphene application on thin film solar cells.

System	Front Contact	Back Contact	Absorber	ETL	HTL
CdTe	Thin-layer graphene (1–5 layers)	Multi-layer graphene with metal	-	-	-
CIGS or CZTS	1–5 layer graphene, need extra surface treatment to decrease R_s_	Multi-layer graphene with metal, need extra surface treatment to decrease R_s_	-	Reduced-GO/TiO_2_ enhanced η.	-
PSC	Thin-layer graphene with interfacial layer for better wettability	Graphene or graphene with metal, need interfacial layer, thickness profile vary	Mix graphene from the initial synthesis	Low concentration with TiO_2_, mixed or separately coated	Need suitable interfacial layer to connect graphene and perovskite
